# Synthetic niclosamide-loaded controlled-release nanospheres with high solubility and stability exerting multiple effects against *Clostridioides difficile*

**DOI:** 10.3389/fmicb.2025.1617631

**Published:** 2025-07-30

**Authors:** Yulei Tai, Meng Zhang, Yuning Han, Hui Hu, Shan Lin, Fangya Zhai, Menglun Tian, Xiaojun Song, Shuangshuang Wan, Yu Chen, Dazhi Jin

**Affiliations:** ^1^School of Laboratory of Medicine, Hangzhou Medical College, Hangzhou, Zhejiang, China; ^2^Laboratory of Biomarkers and In Vitro Diagnosis Translation of Zhejiang Province, Hangzhou, Zhejiang, China; ^3^Department of clinical laboratory, Hangzhou Medical College, Zhejiang Provincial People’s Hospital (Affiliated People’s Hospital), Hangzhou, Zhejiang, China

**Keywords:** loaded controlled-release nanospheres, Niclosamide, *Clostridioides difficile*, spore germination, biofilm formation, multiple effects

## Abstract

**Introduction:**

Niclosamide (NIC) has significant potential as a clinical therapeutic agent for *Clostridioides difficile* infection (CDI); however, its strong hydrophobicity hampers its oral bioavailability, and its active effects against *C. difficile* remain unclear.

**Methods:**

Niclosamide-loaded controlled-release hyaluronic acid-modified poly (lactic-*co*-glycolic acid) naosphernes (NIC@PLGA-HAs) were synthesized using an oil-in-water emulsion technique and their effects on *C. difficile* cell growth, spore germination, biofilm formation, and NIC interaction sites with *C. difficile* toxin B (TcdB) were analyzed.

**Results:**

NIC@PLGA-HAs exhibited enhanced solubility and stability, with a water contact angle on a hydrophilic surface of 65.1° and a zeta potential of 31.57 ± 2.08 mV, and pH-responsive (pH 7.4) controlled-release characteristics compared to free NIC. The NIC@PLGA-HAs killed *C. difficile* vegetative cells at a minimum inhibitory concentration (MIC) of 4 μg/mL. When *C. difficile* cells were treated with NIC@PLGA-HAs at the 1/4 MIC, spore germination and biofilm formation were significantly inhibited compared to those in untreated cells (*P* < 0.01). NIC was found to interact with the receptor-binding domain of TcdB at 24 amino acid sites via an enthalpy-driven reaction (enthalpy change, 36.21 kJ/mol and entropy change, 212.9 J⋅mol/K). *In vivo* experimental findings in Mongolian gerbils indicated that NIC@PLGA-HAs outperformed free NIC in reducing pathological damage, diarrhea severity, weight loss, and TcdB production and enhanced the survival rate.

**Conclusion:**

These findings presented the therapeutic potential of NIC@PLGA-HAs with high solubility and stability, which simultaneously exerted multiple biological activities against *C. difficile*.

## Graphical Abstract

NIC@PLGA-Has present multiple effects against *Clostridioides difficile*. 
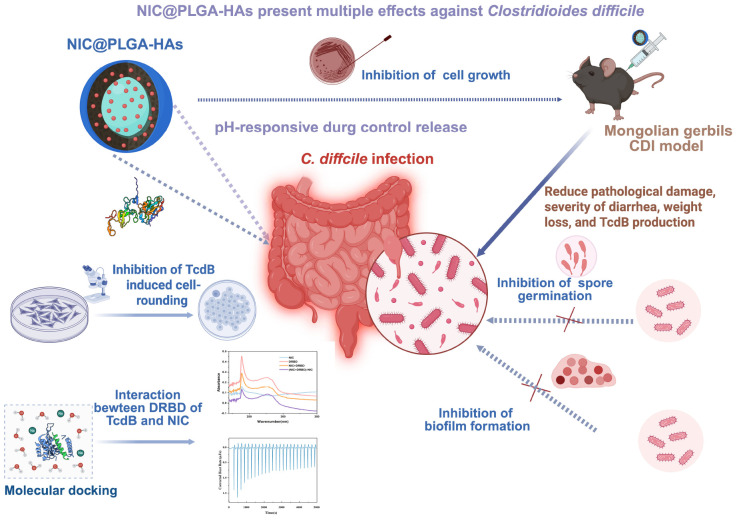

## 1 Introduction

*Clostridioides difficile* is a gram-positive spore-forming bacterium that produces toxin A, toxin B (TcdB), and binary toxin, leading to *C. difficile* infection (CDI) (Paparella et al., 2021). In patients who have used broad-spectrum antibiotics (Rupnik et al., 2009), immunosuppressants (Varma et al., 2022), or chemotherapy drugs (Crobach et al., 2023) for a long time, the destruction of the normal intestinal flora facilitates the invasion, colonization, and rapid multiplication of *C. difficile*. CDI symptoms include diarrhea, pseudomembranous colitis, intestinal perforation, toxic megacolon, and septic shock, which are life-threatening (Sandhu and McBride, 2018). In 2019, *C. difficile* was classified as a high-priority resistant organism by the United States Centers for Disease Control and Prevention (Smits et al., 2016; Centers for Disease Control and Prevention, 2019). The global burden of CDI underscores the urgent need for effective countermeasures (Smits et al., 2016; Centers for Disease Control and Prevention, 2019).

Metronidazole (Johnson et al., 2014) and vancomycin (Louie et al., 2011) are the primary antibiotics recommended for CDI treatment; however, the treatment failure and recurrence rates are high, partially because of gut microbiota disruption (Kelly and LaMont, 2008). Fidaxomicin, a new anti-clostridial agent, shows a similar therapeutic effect but has a lower recurrence rate and fewer treatment-related adverse events than vancomycin (Koon et al., 2018; Diaz-Pollan et al., 2023). However, the use of fidaxomicin also leads to gut microbiota disruption, making CDI treatment more challenging than ever (Tannock et al., 2010). Recently, therapeutic antibodies such as bezlotoxumab (Wilcox et al., 2017) and monoclonal (Lowy et al., 2010) or polyclonal (Steele et al., 2013) anti-*C. difficile* antibodies have been developed to neutralize *C. difficile* toxins. These antibodies play a crucial role in enhancing immune responses, reducing inflammation, restoring gut microbiota, and preventing CDI recurrence (Kelly et al., 2020), and act synergistically with antibiotics and fecal microbiota transplants (Drekonja et al., 2015; Hocquart et al., 2018). Despite their promising potential, several key challenges persist, such as low antibody specificity (Lawry et al., 2018), high production costs (Ooijevaar et al., 2018), potential immune responses (Madan and Petri, 2012), and uncertainties regarding optimal dosing and administration (DuPont, 2013).

Niclosamide (NIC), a United States Food and Drug Administration-approved anthelmintic drug for tapeworm infections (Xu et al., 2020), exhibited multifaceted mechanisms beyond its primary use (Chen et al., 2018). Although its mode of action remains incompletely characterized, known mechanisms included uncoupling of oxidative phosphorylation (Berube et al., 2018). Over recent years, it has been demonstrated that NIC was a multifunctional drug capable of inhibiting or regulating multiple signaling pathways and biological processes (Biersack, 2024). Its therapeutic potential extended beyond parasitic diseases to include (Wardle et al., 2024) cancers, bacterial and viral infections, and metabolic disorders (Berube et al., 2018; Chen et al., 2024). Previous studies indicated the ability of (Singh et al., 2022) NIC and related *salicylanilide* derivatives to inhibit *Mycobacterium tuberculosis* growth. NIC protected RAW264.7 macrophages and CHO cells from anthrax lethal toxin, as well as from *Pseudomonas* exotoxin and diphtheria toxin (Nestorovich and Bezrukov, 2014). Its mechanism may involve endosome (Wang et al., 2022) acidification, inhibition of the Gram-negative pathogen *Pseudomonas aeruginosa*, suppressing its quorum sensing response and production of the signaling molecule acyl homoserine lactone (Costabile et al., 2015). Transcriptomic analysis revealed NIC affected approximately 250 *P*. *aeruginosa* genes, with high specificity for quorum sensing-dependent targets (Francesco et al., 2013). It also suppressed surface motility, reduced secretion of virulence factors (elastase, pyocyanin, and rhamnolipids), and inhibited biofilm formation (Francesco et al., 2013). Oxyclozanide, a structural analog, demonstrated efficacy against the Gram-positive bacterium methicillin-resistant *Staphylococcus aureus* and exhibited bactericidal activity (Li et al., 2023). NIC inhibited severe acute respiratory syndrome coronavirus replication and protected Vero E6 cells from virus-induced cytopathic effects (Li et al., 2020). It has been also demonstrated that NIC was used as a potent, low-micromolar inhibitor of pH-dependent human rhinoviruses and influenza virus, Chikungunya virus, and Zika virus. In recent year, NIC has emerged as a potential candidate for repurposing owing to its safety profile and efficacy in treating CDI by protecting cells from the cytotoxic effects of all three *C. difficile* toxins (Zhang et al., 2022). NIC has been shown to improve symptoms related to both primary infection and recurrence in mouse models (Needham, 2024) and to preserve gut microbiota diversity and composition (Tam et al., 2018). However, the clinical application of NIC remains limited primarily due to two key factors: first, its inherent hydrophobicity substantially reduces bioavailability, especially following oral administration (Wahid et al., 2017; Meng et al., 2020; Liu et al., 2021); and second, the comprehensive biological activity of NIC against *C. difficile* has not yet been fully elucidated (Tam et al., 2018).

Recent advancements in the field of polymer-based oral drug delivery systems may hold the key to overcoming the low hydrophobicity of NIC (Fonte et al., 2015). Poly (lactic-*co*-glycolic acid) (PLGA) (Kumar et al., 2022), a prominent hydrophobic polymer, is widely used in drug delivery systems because of its biocompatibility (Chen et al., 2017) and biodegradability (Chatterjee and Chanda, 2022). Hyaluronic acid (HA) has emerged as a highly promising biopolymer for constructing drug delivery carriers owing to its inherent biocompatibility, non-immunogenicity, hydrophilicity, and bioresorbability (Samiraninezhad et al., 2023). Both PLGA and HA have been extensively studied (Martins et al., 2018). Our previous study demonstrated that hydrophilic HA-modified PLGA-HA microspheres, synthesized via microfluidic technology, exhibited sustained release behavior at pH 5.0 and 7.4, but not at pH 2.0 (Tai et al., 2023). The HA modification significantly enhanced the solubility of NIC and augmented its biological activity, thereby addressing the need for effective intestinal drug delivery carriers (Tai et al., 2023). The microfluidic synthesis of polymeric nano-delivery systems entails the optimization of reactant flow rates, selection of suitable polymers, and fine-tuning of synthesis conditions to achieve uniform particle size, stability, and efficient cargo encapsulation (Ran et al., 2017; Zhang et al., 2023).

In the present study, PLGA and HA were used to synthesize NIC-loaded nanospheres using the oil-in-water emulsion technique to assess the enhancement of the water solubility of NIC facilitated by the PLGA-HA nano-delivery system, and the biological activities of NIC@PLGA-HA nanoparticles (NIC@PLGA-HAs) against *C. difficile* were explored using *in vivo* and *in vitro* experiments.

## 2 Materials and methods

### 2.1 Reagents and cell lines

PLGA (50:50 mass ratio of lactic acid to glycolic acid, Mw 24,000–38,000, 76,000–115,000, acid-terminated) and NIC (mass fraction 98%) were purchased from Aladdin Reagent Co. Ltd. HA (amine-terminated Mw 80,000–150,000 Da) was purchased from Shanghai Macklin Biochemical Technology Co., Ltd. (Shanghai, China). Polyvinyl alcohol [type 105, alcoholysis degree, 98%–99% (mol/mol)] was purchased from Beijing WoKai Biological Technology Co. Ltd. All reagents are of biomedical grade and used as received. Caco-2 human epithelial colorectal adenocarcinoma cells (correctly identified by STR, freeze cells), fetal bovine serum (New Zealand Origin), streptomycin (United States Pharmacopeia Grade), and penicillin (United States Pharmacopeia Grade) were purchased from Shanghai Sangon Biotech Co. Ltd. Cells were cultured in high-glucose Dulbecco’s modified Eagle’s medium (DMEM) supplemented with 10% fetal bovine serum, 100 U/mL penicillin, and 100 μg/mL streptomycin at 37°C with 5% CO_2_ and 95% humidity.

### 2.2 NIC@PLGA-HA synthesis

NIC@PLGA-HA was prepared using the oil-in-water emulsion technique (Tai et al., 2023). First, NIC (2.5, 3.0, and 3.5 mg) and PLGA (25.0 mg) were dissolved in ethyl acetate (5.0 mL), respectively. The solution was mixed with 10 mL of polyvinyl alcohol aqueous solution (1.2%, wt%) and 150 mg of HA until uniform consistency was achieved. Following sonication with the 100, 120, 150, 280, 350 w ultrasonic waves for 5, 10, and 15 min respectively, the mixture was stirred at room temperature until ethyl acetate was completely evaporated. The excess liquid was decanted by filtration, and the sediment was rinsed with deionized water, frozen at −80°C overnight, and lyophilized for 48 h.

### 2.3 NIC@PLGA-HA characterization

The chemical structures of the NIC@PLGA-HAs and their components were analyzed by Fourier Transform infrared spectroscopy (FT-IR, Nicolet iS 5; Thermo, Waltham, MA, United States) and X-ray diffraction (XRD, Bruker D8 Venture; Bruker Co., Germany). The light transmittance of the samples was measured using an Ultraviolet–Visible spectrophotometer (UV-vis, UV2600; Shimadzu, Japan). Their morphologies were observed by Transmission electron microscope (TEM, JEM 2100F; JEOL, Japan). The particle size and zeta potential distribution of the samples were determined using a particle size analyzer (Dynamic light scattering (DLS); Zetasizer Nano ZS90; Malvern Pananytical, Malvern, United Kingdom). The thermal stabilities of the NIC@PLGA-HAs were analyzed using a Diamond thermogravimetry analysis/differential thermal instrument (TG/DTA, PerkinElmer, United States). The hydrophilicity of the samples was determined by contact angle measurements (JY-82C; Chende, China). The detailed process has been previously described (Tai et al., 2023).

### 2.4 Determination of the drug-loading rate and drug release

To determine the drug-loading rate, 10 mg of freeze-dried NIC@PLGA-HAs was added to 5 mL of ethanol and placed in a shaker incubator (37°C, 100 × *g*) for 24 h until all non-encapsulated NIC was dissolved. After the solution was centrifuged at 14,000 × *g* at 4°C for 15 min, the free NIC absorbance was measured at 370 nm using an UV-vis spectrophotometer (UV-2600, Shimadzu). Finally, the concentration of free NIC in the solution was calculated using a calibration curve (A = 0.0594C + 0.0419, *R*^2^ = 0.9966), and the loading efficiency was calculated (Tai et al., 2023). To assess the drug release behavior, 10 mg of NIC and NIC@PLGA-HA were dispersed in 3 mL of release medium buffered solution. The solution was enclosed in dialysis bags (12,000 Da) and immersed in buffer solution (100 mL) in a shaking incubator (37°C, 100 × *g*). The cumulative release percentage was calculated as described in our previous report (Tai et al., 2023).

### 2.5 Measurement of the minimum inhibitory concentration (MIC) of NIC@PLGA-HAs

The MIC of NIC@PLGA-HAs were determined using an agar dilution assay according to the Clinical and Laboratory Standards Institute guidelines (Lewis, 2024). Three *C. difficile* standard strains [American Type Culture Collection (ATCC) BAA-1870, 700057, and 43255] and four clinical isolates were spotted three times on Brucella agar supplemented with hemin (5 μg/mL), vitamin K (1 μg/mL), and defibrillated sheep blood (5%, v/v) in the presence of NIC or NIC@PLGA-HA at concentrations ranging from 1 to 128 μg/mL using 2-fold serial dilutions. Vancomycin at concentrations ranging from 1 to 16 μg/mL using 2-fold serial dilutions was used as positive controls (Tam et al., 2018). The plates were incubated in GENbag Anaer (BioMérieux, Marcy l’Étoile, France) in an anaerobic chamber at 37°C for 48 h. MICs were determined in duplicate and were reported as the lowest concentration at which no colonies were observed.

### 2.6 Measurement of *C. difficile* biofilm formation

*C. difficile* biofilms were measured as reported previously (Dapa and Unnikrishnan, 2013), with minor modifications. An overnight culture of *C. difficile* cells (ATCC BAA-1870) was diluted to 1 × 10^6^ colony-forming units (CFU)/mL in brain heart infusion-supplemented (BHIS) medium supplemented with 10 g/L glucose and 50 mM sodium phosphate buffer (pH 7.5). Then, 960 μL of the dilution was transferred into 24-well plates and incubated under anaerobic conditions at 37°C for 24 h. Then, 40 μL of BHIS with or without the 1/4 MIC of vancomycin, NIC, or NIC@PLGA-HA was added to the wells and incubated under anaerobic conditions at 37°C for 24 h. After that, the culture supernatant was removed, the wells were washed once with PBS and stained for 30 min with 0.1% (wt/vol) crystal violet in water. After the wells were washed twice with PBS, the bound crystal violet was solubilized by 95% ethanol. The absorbance at 570 nm was determined with a Synergy HT microplate reader (BioTek, CA, United States). Experiments were independently repeated three times.

### 2.7 Determination of *C. difficile* spore germination

*C. difficile* spores (ATCC BAA-1870) were prepared and purified as described previously (Sorg and Sonenshein, 2010). Briefly, 10^7^ spores/mL suspended in BHIS medium supplemented with 0.1% taurocholate was mixed without and with the 1/4 MIC of NIC or NIC@PLGA-HAs and incubated in an anaerobic environment at 37°C for 48 h. At 2 days intervals from days 2 to 14, the culture was inoculated onto BHIS agar plates for further subculturing in an anaerobic environment at 37°C for 48 h, respectively. CFUs on each plate were counted. Experiments were independently repeated three times.

### 2.8 TcdB toxicity assay

*C. difficile* toxin B toxicity was performed as previously described (Tam et al., 2018). Caco-2 cells were seeded in a 24-well plate at 5 × 10^4^ cells/well and cultured for 24 h. After mixing for 15 min, NIC@PLGA-HAs (10 ng/mL) and purified TcdB (1.6 ng/mL) prepared in our laboratory (Lin et al., 2024) were added to the wells. Purified TcdB alone at the same concentration was used as the positive control. The viability of Caco-2 cells was assessed at 12, 24, and 48 h using the CellTiter-Glo^®^ Luminescent Cell Viability Assay (Promega, Beijing, China). Morphologically, rounded cells were counted and calculated relative to the total number of cells.

### 2.9 Molecular modeling and kinetic simulation

Molecular docking simulation was conducted using AutoDock 4.2 by the Lamarckian genetic algorithm. The structure of the NIC was generated using KingDraw and optimized using the Gaussian 09 program. Geometry optimization was performed using the hybrid Becke, 3-parameter, Lee-Yang-Parr functional together with the transmembrane delivery and receptor-binding domain (DRBD) C, H, O, N, and F atoms. The crystal structure of DRBD (PDB ID: 4nc2) was obtained from Brook-Haven Protein Bank.^1^ Molecular dynamics simulation was employed to further refine the binding mode of the molecule-protein complex using the Desmond program. Both the protein and the molecule were parameterized using the OPLS4 force field and the SPC/E model was used as the water solvent. The molecule-protein complex was positioned within a cubic water box and solvated (Decherchi and Cavalli, 2020). Furthermore, for AlphaFold 3-specific docking, both the protein sequence and ligand structure were inputted into AlphaFold 3. The model predicted the bound complex by integrating ligand atomic coordinates and calculating binding affinity scores (Abramson et al., 2024; He et al., 2025).

### 2.10 Expression and purification of wide type and mutant of TcdB DRBD

Recombinant proteins were expressed and purified as previously reported (Chen et al., 2021). Gene sequences corresponding to TcdB DRBD with wide type (DRBD^WT^) and mutant (DRBD^MT^) were cloned into the pET-28a (+) vector (Sangon Biotech Co., Ltd.). DRBD^MT^ was mutated in the amino acids as predicted above according to binding energies. Proteins expressed with a 6-His tag at the N-terminus were purified using Ni^2+^-NTA affinity chromatography (Sangon Biotech Co., Ltd.).

### 2.11 Ultraviolet-visible (UV-vis) absorption spectroscopy and isothermal titration calorimetry

Ultraviolet-visible spectra were recorded using an UV-vis spectrophotometer (UV2600, Shimadzu, Japan) to measure the interaction between DRBD and NIC in the wavelength range of 200–800 nm at room temperature (Wang et al., 2019). Isothermal titration calorimetry (ITC) was conducted on a NANOITC (TA Instruments, United States) at 25°C in a well containing 200 μL of reaction mixture (50 mM PBS, pH 7.4, 15% DMSO) and 12 μM TcdB-DRBD. The ligand was titrated into the protein solution over 25 injections of 2 μL for 6 s, with a 150 s equilibration period between injections. Data fit the model of a distinct binding site. The first injection for each sample was excluded from data fitting. A separate titration without TcdB-DRBD was performed for each condition to subtract the background heat of dilution (Gurung et al., 2017).

### 2.12 Animal model

The primary CDI model was established according to our previous report (Wan et al., 2024). Mongolian gerbils were purchased from the Animal Center of the Hangzhou Medical College. Mongolian gerbils were divided into five groups (*N* = 10 per group) and received mixed antibiotics in drinking water for 3 days. Animals were further treated with clindamycin (10 mg/kg) for 1 day. Mongolian gerbils in the four infected groups were orally administered 10^5^ CFU of *C. difficile* spores (ATCC BAA-1870), and those in the non-infected control group were administered water with 5% DMSO. Animals in the three infection groups were treated with vancomycin, NIC, or NIC@PLGA-HA (20 mg/kg suspended in drinking water with 5% DMSO). The animals were monitored for signs of disease such as diarrhea, wet tails, and weight loss. After 4 days, the animals were euthanized and stool samples and intestinal tissues were collected. All animal procedures were performed in accordance with the Guidelines for Care and Use of Laboratory Animals of the Zhejiang Institute for Food and Drug Control, and were approved by the Animal Ethics Committee of the Zhejiang Institute for Food and Drug Control. The experimental animal license number is SCXK (Zhe) 2019-0002.

### 2.13 Statistical analysis

Data were statistically analyzed using GraphPad Prism version 10 (GraphPad Software, San Diego, CA, United States). Significant differences were determined using one-way analysis of variance (ANOVA). Statistical significance was set at *P* < 0.05.

## 3 Results and discussion

### 3.1 Characterization of NIC@PLGA-HAs

**FIGURE 1:**
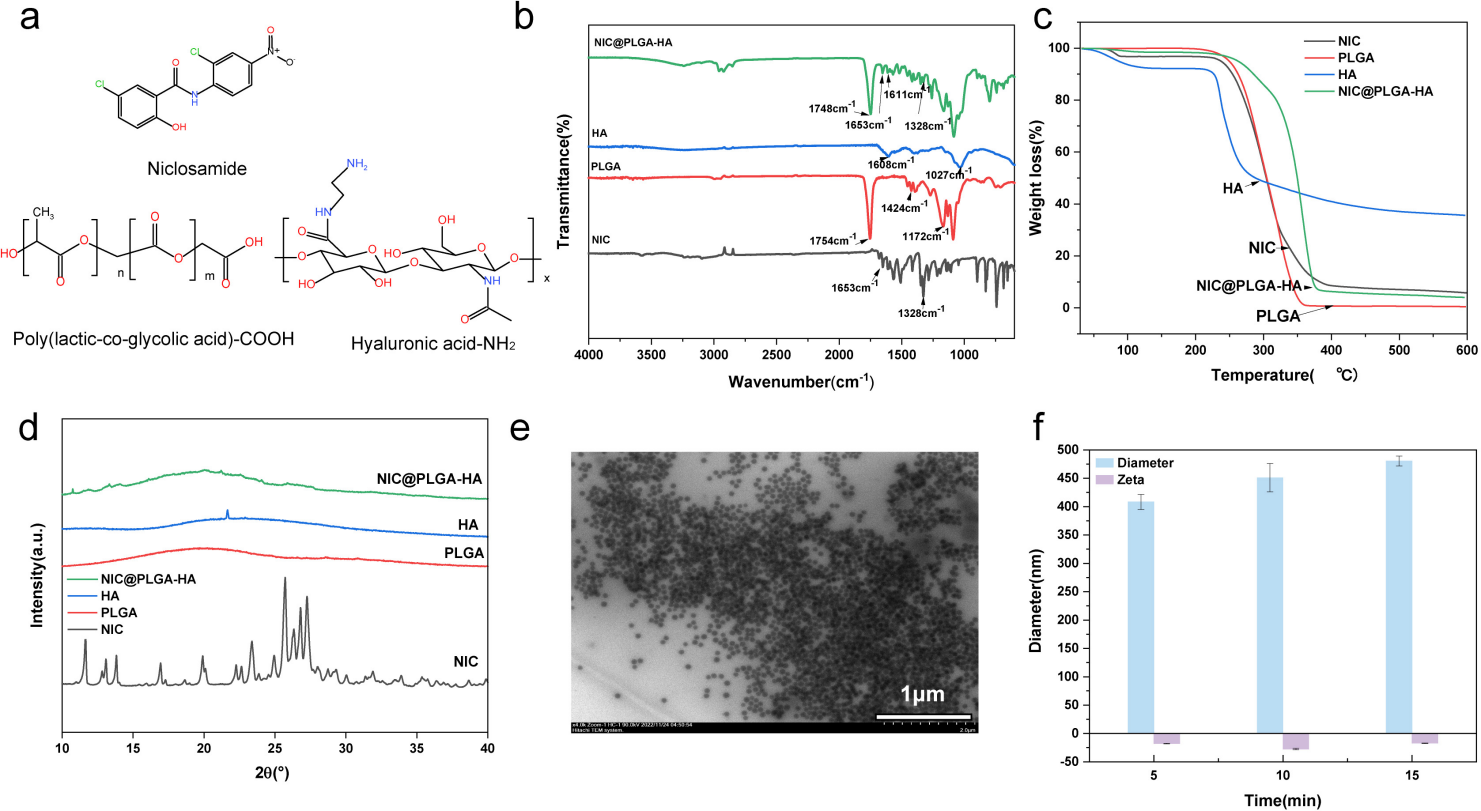
Characterization of NIC@PLGA-HAs. The structural formulas **(a)** of Niclosamide (NIC), Poly (lactic-*co*-glycolic acid) (PLGA), and Hyaluronic acid (HA). Fourier Transform infrared spectroscopy (FT-IR) spectra **(b)**, thermogravimetry (TG) curves **(c)** and X-ray diffraction (XRD) patterns **(d)** of NIC, PLGA, HA and NIC@PLGA-HAs. TEM image **(e)** of NIC@PLGA-HAs. The mean particle diameters and zeta potentials **(f)** of NIC@PLGA-HAs (3.5 mg) synthesized for 5 min under different ultrasonication time.

FT-IR, TEM, DLS, XRD, thermogravimetry (TG), and contact angle measurements were used to analyze the chemical composition, microscopic morphology, particle size, crystal structure, stability, and hydrophilicity of the NIC@PLGA-HAs (Figure 1). The chemical structures of NIC, PLGA, and HA are shown in Figure 1a. Compared with those of NIC, the FT-IR spectra of the NIC@PLGA-HAs exhibited two distinct absorption peaks for NIC at 1,328 and 1,653 cm^–1^, corresponding to the C-N and -C = O functional groups, respectively, indicating that NIC was successfully loaded into the PLGA-HAs (Figure 1b). Compared with those of PLGA, the FT-IR spectra of NIC@PLGA-HAs featured peaks at 1,748, 1,424, 1,172, and 1,611 cm^–1^, corresponding to the stretching vibrations of C = O, C-H, and C-O-C functional groups of PLGA and O-H, N-H, and C-O functional groups of HA, respectively, indicating that the PLGA-HA polymeric shell encapsulated the NIC.

Thermogravimetry and derivative thermogravimetry revealed sharp melting point peaks for unprocessed NIC, HA, and PLGA, which appeared at 307.7, 238.2, and 324.3°C respectively, and disappeared for NIC@PLGA-HAs, corroborating that NIC was loaded into the PLGA-HAs (Figure 1c and Supplementary Figure 1). For NIC@PLGA-HAs, a new peak appeared at 361.7°C, whereas that of NIC reduced in size. The peak had a large surface area and close contact between PLGA-HAs and NIC. These changes were considered to indicate a change in the NIC from the crystalline to the amorphous state and an increase in the thermal stability. XRD was used to confirm the crystal structures of NIC, HA, PLGA, and NIC@PLGA-HA (Figure 1d), confirming the amorphous nature of NIC@PLGA-HAs. The disappearance or reduced intensities of the XRD peaks are indicative of the amorphous nature of the nanoparticles compared with the unprocessed NIC.

TEM was used to investigate the micromorphology of NIC@PLGA-HAs with different preparation conditions (Figure 1e and Supplementary Figure 2). TEM images revealed that the NIC@PLGA-HAs maintained dispersibility and a uniform shape, suggesting that the particle size and shape of the NIC@PLGA-HAs were related to the concentration of NIC, ultrasonication time, and power. DLS and zeta potential measurements were straightforward table-top techniques that can be performed in standard laboratory settings to assess the hydrodynamic size and surface charge (Bhattacharjee, 2016). The diameters, mean diameter, size distribution, and polydispersity index of NIC@PLGA-HAs with different preparation conditions were verified using DLS (Figure 1f and Supplementary Figure 3), revealing the homogeneous particle hydrodynamic diameters of 2,533.7 nm for NIC particles and 259–530 nm for NIC@PLGA-HAs, depending on the NIC concentration, ultrasonication time, and power. The reduced particle size in NIC@PLGA-HAs was attributed to the hydrophilic surface of PLGA-HA counteracting NIC’s aggregation tendency caused by its low solubility and hydrophobicity. The polydispersity index value of NIC@PLGA-HAs ranged from 0.2 to 0.4, suggesting a moderate level of polydispersity. The zeta potentials of NIC@PLGA-HA synthesized under different ultrasonication time, the different NIC concentrations, and different powers were also measured (Figure 1f and Supplementary Figure 3). In principle, a system was considered electrostatically stable if the absolute zeta potential was above 20 mV (Chatterjee et al., 2019). The highest absolute zeta potential of 31.57 ± 2.08 mV was observed for NIC@PLGA-HAs synthesized by adding 3.5 mg NIC to the mixture using an ultrasound assay, as this lowered the probability of particle aggregation. NIC@PLGA-HAs were highly stable in solution, as evidenced by the particle size and zeta potential measurements, rendering them well suited for drug delivery applications. When PLGA and HA were physically mixed to form emulsified particles, the presence of HA may affect the final particle zeta potential (Hu et al., 2015). HA was a negatively charged polysaccharide, with the carboxyl groups on its molecular chains providing negative charges (Naik et al., 2017). When combined with PLGA (through physical adsorption or encapsulation within PLGA particles), the surface negative charges of HA increased the overall negative charge of the particles, thereby enhancing the zeta potential. Generally, the as-prepared NIC@PLGA-HAs exhibited high drug loading, a narrow size distribution, and both colloidal and thermal stability, demonstrating potential for manufacturability.

### 3.2 Hydrophilicity and NIC release behavior of NIC@PLGA-HAs

**FIGURE 2:**
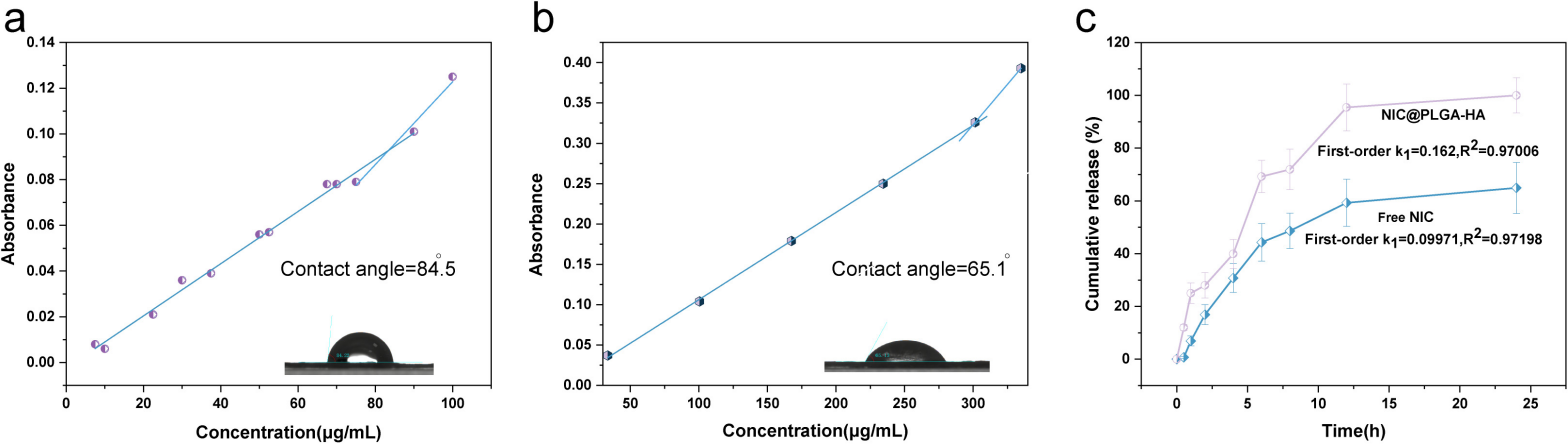
Water-solubility, stability and drug release behavior of NIC@PLGA-HAs. Contact angle images and critical micelle concentrat (CMC) curves of **(a)** NIC and **(b)** NIC@PLGA-HAs, and **(c)** *in vitro* drug release at pH 7.4 and 37°C. NIC@PLGA-HAs were prepared with the Niclosamide (NIC) (3.5 mg) (mean ± SD, *n* = 3).

The water contact angle of NIC@PLGA-HAs was 65.1°, which was less than that of hydrophobic NIC (84.5°) (Figures 2a, b), demonstrating that PLGA-HA copolymers improved the hydrophilicity of NIC by connecting with other water-soluble substances, which was in agreement with previous findings (Naik et al., 2017; Wu H. et al., 2020). The critical micelle concentration (CMC) of NIC@PLGA-HAs was determined using a probe-free UV-vis spectroscopy (Ma et al., 2016). The CMCs of NIC and NIC@PLGA-HAs were shown in Figure 2. Through linear regression analysis, CMCs was determined to be 83.23 μg/mL for NIC and 300.86 μg/mL for NIC@PLGA-HAs. Upon exceeding the CMC, NIC@PLGA-HAs self-assembled into micelles in an aqueous medium. The hydrophobic nature of the NIC component within NIC@PLGA-HAs drove them to evade the aqueous environment, contributing to the formation of a hydrophobic micellar core. Therefore, an increase in the NIC concentration resulted in a corresponding increase in UV absorbance. Notably, the CMC of NIC@PLGA-HAs was higher than that of NIC, which could be attributed to the encapsulation by PLGA-HA. The increased CMC value indicated enhanced hydrophilicity (Ma et al., 2016), which aligned with the contact angle measurement results.

*In vitro* drug release from NIC@PLGA-HAs synthesized using the microfluidic method was pH-dependent, in line with our previous study (Tai et al., 2023). NIC releasing from PLGA-HAs was faster at pH 7.4 than at pH 5.0 and 2.0, demonstrating that NIC@PLGA-HAs had the pH-dependent nature of drug release and good solubility of PLGA-HA at the high pH, as reported previously (Martins et al., 2018). PLGA-HA had a primary carboxyl group and an ester group, which became deprotonated at pH 7.4, inducing the formation of soluble biopolymeric hydrogel networks (Wu H. et al., 2020). NIC@PLGA-HAs exhibited rapid burst release during the first 8 h, suggesting no chemical interactions between NIC and the PLGA-HA polymeric chains. After 8 h, the percentage of NIC released from PLGA-HAs was significantly higher than that of free NIC, and the release from NIC@PLGA-HAs was highly consistent with a quasi-first-order kinetic model (*R*^2^ = 0.97) (Figure 2c), which suggested that NIC adsorption occurred primarily via chemical or physical adsorption. The surface modification of PLGA nanoparticles with HA has numerous advantages, such as reducing the burst effect in drug release, facilitating zeta potential inversion for enhanced cellular adhesion and retention at the target site (Chen et al., 2017), and providing the opportunity to conjugate targeting ligands to surface amino groups (Chatterjee and Chanda, 2022). Therefore, this modification was beneficial to the binding between NIC and TcdB proteins, thereby likely improving the CDI treatment effect. Furthermore, the incorporation of HA enabled pH-dependent drug release, whereas PLGA-HA-modified nanoparticles offered dual protection against enzymatic and gastric degradation and reduced the gastrointestinal irritation caused by NIC via a controlled release mechanism (Chatterjee et al., 2019).

### 3.3 NIC@PLGA-HAs inhibit *C. difficile* vegetative cell growth, spore germination, and biofilm formation

The anti-clostridial activity of NIC@PLGA-HAs was evaluated using seven *C. difficile* strains (Table 1). NIC@PLGA-HAs inhibited the growth of all *C. difficile* strains tested, at concentrations ranging from 0.25 to 4 μg/mL. The half-maximal inhibitory concentration (IC_50_) was 1 μg/mL and the 90% inhibitory concentration (IC_90_) was 4 μg/mL. The minimal inhibitory concentration (MIC) of vancomycin ranged from 0.25 to 2 μg/mL (IC_50_ and IC_90_: 0.5 and 1 μg/mL). Therefore, the inhibitory capability of NIC@PLGA-HAs was comparable to that of vancomycin, the preferred therapeutic agent for primary and severe CDI (Hastey et al., 2017). However, the potential for prolonged NIC@PLGA-HAs exposure to induce resistance associated gene mutations remains experimentally unaddressed. The NIC@PLGA-HAs’ multi-component nature theoretically reduced selective pressure for drug resistance, but which should be further verified later (Su et al., 2021).

**TABLE 1:** Minimum inhibitory concentratio (MIC) of three agents for inhibiting growth of *C. difficile*.

Strain	Agent (μg/mL)		
	Vancomycin	NIC	NIC@PLGA-HAs
*C. difficile* (ATCC BAA-1870)	2–4	4	2–4
*C. difficile* (ATCC 700057)	1	8	4
*C. difficile* (ATCC 43255)	1	16	4
*C. difficile* (clinical isolate, ST35^a^)	1	4	2
*C. difficile* (clinical isolate, ST54)	1	4-8	0.5
*C. difficile* (clinical isolate, ST39)	1	4	1–2
*C. difficile* (clinical isolate, ST37)	2	4	4

^a^ST, sequence type.

**FIGURE 3:**
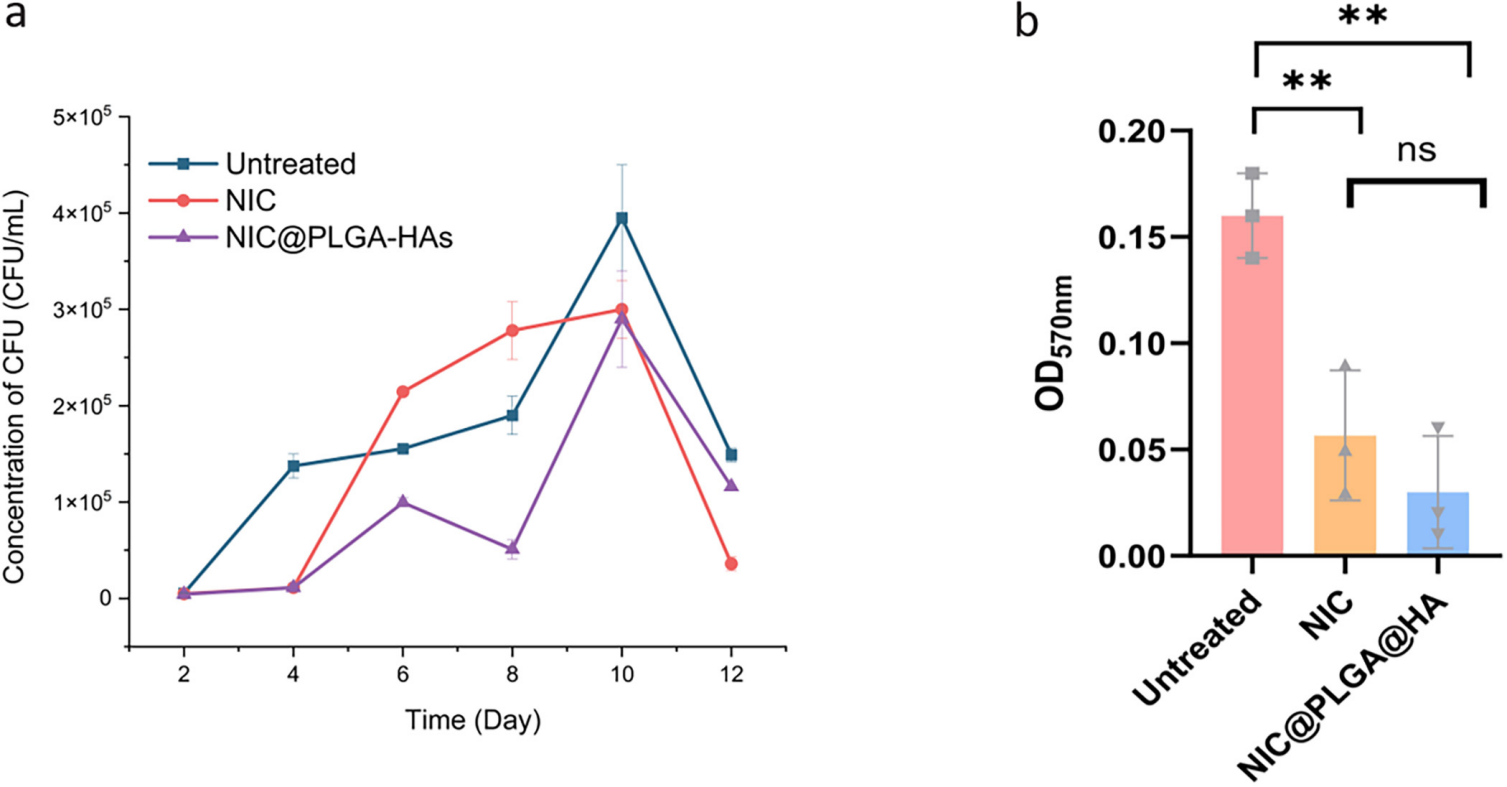
NIC@PLGA-HAs inhibited spore germination and biofilm formation of *C. difficile*. **(a)** NIC@PLGA-HAs inhibited spore germination at the 1/4 minimal inhibitory concentration (MIC) compared to untreated *C. difficile*. **(b)** Biofilm production measured by crystal-violet staining absorbance at 570 nm. The biofilm production was significantly inhibited by NIC@PLGA-HAs in comparison to untreated *C. difficile* (ns, *P* > 0.05; **P < 0.01).

*C. difficile* spore germination was a major factor in treatment failure of CDI and CDI recurrence (Zhu et al., 2018). Inhibition of *C. difficile* spore germination contributed to the effectiveness of CDI treatment and reduced CDI recurrence (Daou et al., 2019). In contrast to NIC, NIC@PLGA-HAs at the 1/4 MIC were able to inhibit *C. difficile* spore germination consistently 10 days after exposure (Figure 3a). However, *C. difficile* spore germination was distinctly decreased on the 12^th^ day after exposure or not to NIC and NIC@PLGA-HAs at the 1/4 MIC, which phenomena should be further investigated. Overall, these results demonstrated that NIC@PLGA-HAs exhibited maximal inhibitory efficacy against *C. difficile* spore germination specifically during the initial 2–4 days (Semenyuk et al., 2014), suggesting heightened sensitivity to the outgrowth stage of spore germination (Horvat and Rupnik, 2018).

*C. difficile* biofilms were closely served as a niche for *C. difficile* spore formation (Barra-Carrasco and Paredes-Sabja, 2014). Additionally, *C. difficile* biofilm enhanced its persistence in the gut, increased its resistance to antibiotics, promoted CDI recurrence, and prolonged the duration of CDI (Stewart et al., 2020; Dicks, 2023). *C. difficile* treated with NIC or NIC@PLGA-HA at the 1/4 MIC produced significantly less biofilm than untreated *C. difficile* (*P* < 0.01), and no significant difference was found between NIC and NIC@PLGA-HA (Figure 3b).

### 3.4 NIC@PLGA-HAs bind to the delivery and receptor-binding domain (DRBD) domain of TcdB to suppress cytotoxicity

**FIGURE 4:**
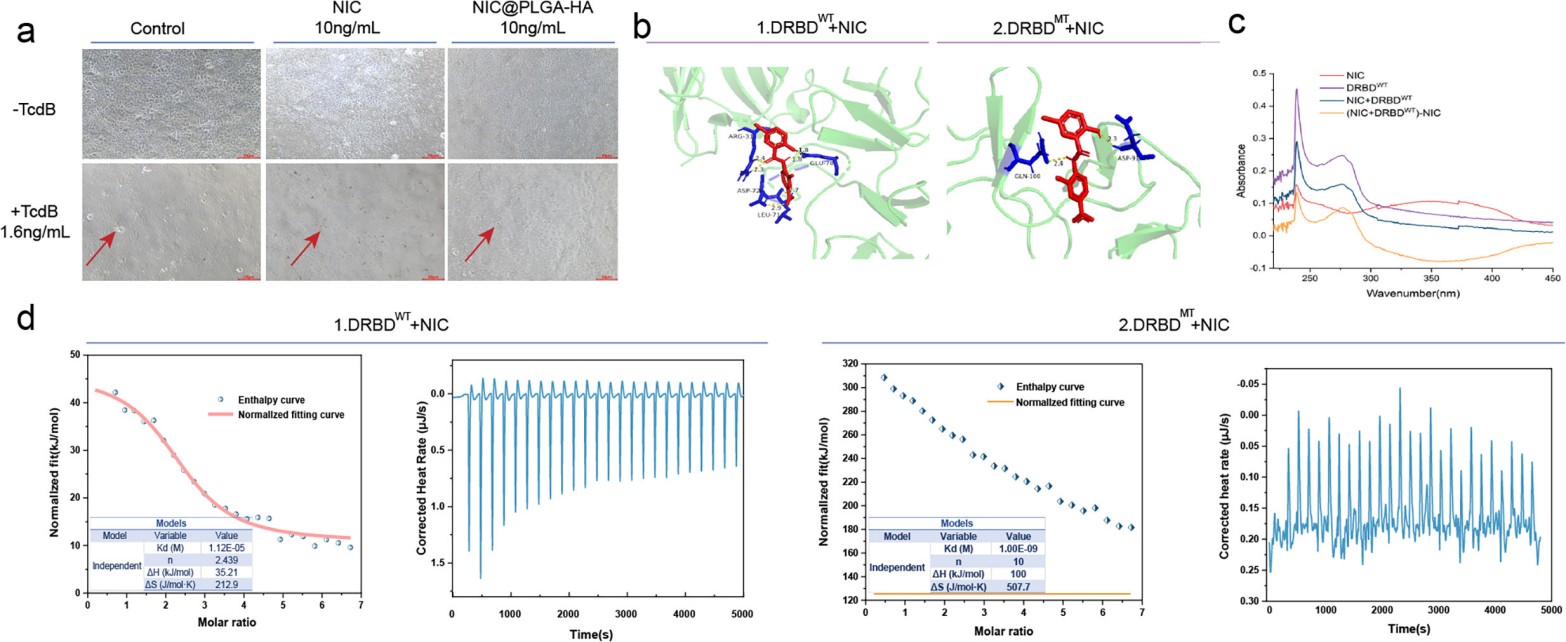
Interaction between delivery and receptor-binding domain (DRBD) in *C. difficile* toxin B (TcdB) and Niclosamide (NIC). **(a)** NIC against TcdB-mediated Caco-2 cell rounding. **(b)** Views of the interaction between each of TcdB, DRBD, and mutated DRBD and NIC (electrostatic surface). **(c)** Ultraviolet-visible (UV-vis) monitoring curves when NIC was interacted with DRBD. **(d)** Isothermal titration calorimetry (ITC) curves when NIC was interacted with DRBD and DRBD mutants, respectively.

Effective treatment should target not only *C. difficile* cells and spores but also their toxins (Nooij et al., 2024; Tiseo et al., 2024). *C. difficile* toxins, particularly TcdB, damaged the colonic epithelium, leading to inflammation and severe diarrhea (Lyras et al., 2009). TcdB contained four structural domains, of which the DRBD recognized receptors on intestinal cells and assisted the glycosyltransferase domain to enter host cell cytosol (Kuehne et al., 2010). CDI has been effectively treated by blocking the interaction between DRBD of TcdB and host cell surface receptors (Dicks, 2023). Our previous study demonstrated that at a low concentration (10 ng/mL), NIC@PLGA-HA did not inhibit the growth of Caco-2 cells (Tai et al., 2023). The present study showed that TcdB (1.6 ng/mL)-induced cell rounding of Caco-2 cells was inhibited in the presence of NIC@PLGA-HA (10 ng/mL) (Figure 4a).

Based on the molecular docking between NIC and TcdB DRBD^WT^, DRBD^MT^ was constructed by artificially mutating these amino acids (Supplementary Table 1). Nucleotide sequences with gene mutations were produced using the overlap PCR with the primers listed in (Supplementary Table 2). Fractions containing wild-type DRBD^WT^ and mutant DRBD^MT^ were verified by SDS-PAGE (Supplementary Figure 4). To gain a deeper insight into the binding mechanism between DRBD of TcdB and NIC, we simulated the molecular docking between NIC and TcdB DRBD^WT^ and DRBD^MT^, respectively (Figure 4b). NIC entered the cavity of TcdB, and 24 amino acids (Table 2) spatially complemented the structure of NIC, thereby stabilizing the conformation of the resultant complex (Figure 4b-1) as described previously (Lawal et al., 2023). This association was characterized by the hydrogen bonding of hydrogen atoms within the molecule at distances of 2.9Å, 2.7Å, 2.4Å, 2.3Å, 1.8 Å, and etc. Hydrophobic and electrostatic interactions played a critical role in the binding between DRBD and NIC. The variance in residues from the DRBD that tethered to the NIC elucidated the observed diversity in binding outcomes. The results showed that NIC interacted unusually with the DRBD^MT^, forming an unstable complex compared to TcdB DRBD^WT^ (Figure 4b-2). The AlphaFold3 was also used to further confirm the interaction binding sites. The results showed that NIC bound to significantly more sites on TcdB than those on mutated TcdB and exhibited higher binding energy (−6.30 Kcal/mol) (Supplementary Figure 5). The UV absorption spectra of NIC interacting with DRBD under simulated physiological conditions were shown in Figure 4c. Complex formation between DRBD and NIC induced clear peak changes at 275 and 230 nm compared to those for DRBD and NIC alone, confirming successful complexation. In the ITC analysis, the energy change curves indicated an endothermic process during DRBD titration with NIC. The endothermicity of the peaks decreased with continuous DRBD-NIC combination, displaying a gradual saturation process because of heat adsorption by the complex (Figure 4d). To eliminate interference from dilution heat in the curve-fitting process, an NIC DMSO solution was titrated into a PBS solution. The titration reactions indicated an endothermic dilution process. In contrast, the fitting results of the ITC curves obtained under identical experimental conditions for DRBD^MT^ and NIC failed to demonstrate notable binding features, suggesting weak or absent binding interactions (Figure 4d). According to the Wiseman isotherm theory (Wang et al., 2019), an S-shaped fitted curve indicated strong interactions, whereas a parabolic curve suggested low affinity. Therefore, the interaction between the NIC and DRBD^WT^ was stronger than between the NIC and DRBD^MT^.

**TABLE 2:** Interaction sites between Niclosamide (NIC) and DRBD^WT^/DRBD^MT^ simulated by the molecular docking.

Proteins	Binding energy (Kcal/mol)	Amino acid residues of binding sites	Length of hydrogen bonds (Å)
DRBD	−6.22	ILE-4, ASN-2, ARG-31, ASP-72	2.8, 2.1, 3.5, 2.3
		ASP-112, ARG-115, SER-51, TYR-38	3.2, 2.7, 2.6, 2.5
		THR-101, ASP-152, GLU-781, SER-153	2.3, 1.8, 3.5, 3.2
		LYS-454, SER-470, ASN-472, ASP-431	1.7, 3.1, 2.1, 2.2
		LYS-231, ALA-226;ASN-2	2.7, 1.5; 3.0
		TYR-424, THR-449, ASN-448, ASN-276, and LYS-259	2.9, 3.3, 3.4, 1.8, and 1.9
Mutated DRBD	−1.89	ASN-276 and LYS-259	1.8 and 1.9

The thermodynamic parameters for the interaction between NIC and DRBD were listed in Table 2. The negative Gibbs energy change value (ΔG, −27.26 kJ/mol) indicated that the interaction between NIC and DRBD was a spontaneous process. NIC displayed the strongest binding affinity with DRBD, with a binding energy of −6.22 Kcal/mol. The low ΔG value of the NIC–DRBD complex indicated these interactions. The positive values of ΔH (36.21 kJ/mol) and ΔS (212.9J mol/K) supported that the binding between NIC and DRBD was an enthalpy-driven reaction (Figure 4d). Although this process required energy absorption, an increase in the entropy caused it to occur spontaneously. In biological processes, such phenomena were often involved in protein folding, molecular binding, or other intermolecular forces, and are typically accompanied by a transition from a structured to a less ordered state, naturally gravitating toward a state of equilibrium (Huang and Zhan, 2007).

Together, the above results indicated that the interaction between NIC and DRBD was multifaceted and involved hydrophobic forces, as well as hydrogen bonds and electrostatic interactions facilitated by ionic and polar residues (e.g., arginine and asparagine) in the adjacent ligand, contributing to complex stabilization. A previous study demonstrated that NIC inhibited TcdB toxicity by targeting pore formation (Tam et al., 2018). NIC has been documented to moderately elevate endosomal pH via a unique proton-shuttle mechanism, distinguishing it from other methods of endosomal deacidification, such as lysosomotropism (Jurgeit et al., 2012). Our findings suggested that NIC not only inhibited spore germination and biofilm formation, but also prevented TcdB-induced cell rounding by binding to the DRBD of TcdB.

### 3.5 *In vivo* treatment of an animal CDI model with NIC@PLGA-HAs

**FIGURE 5:**
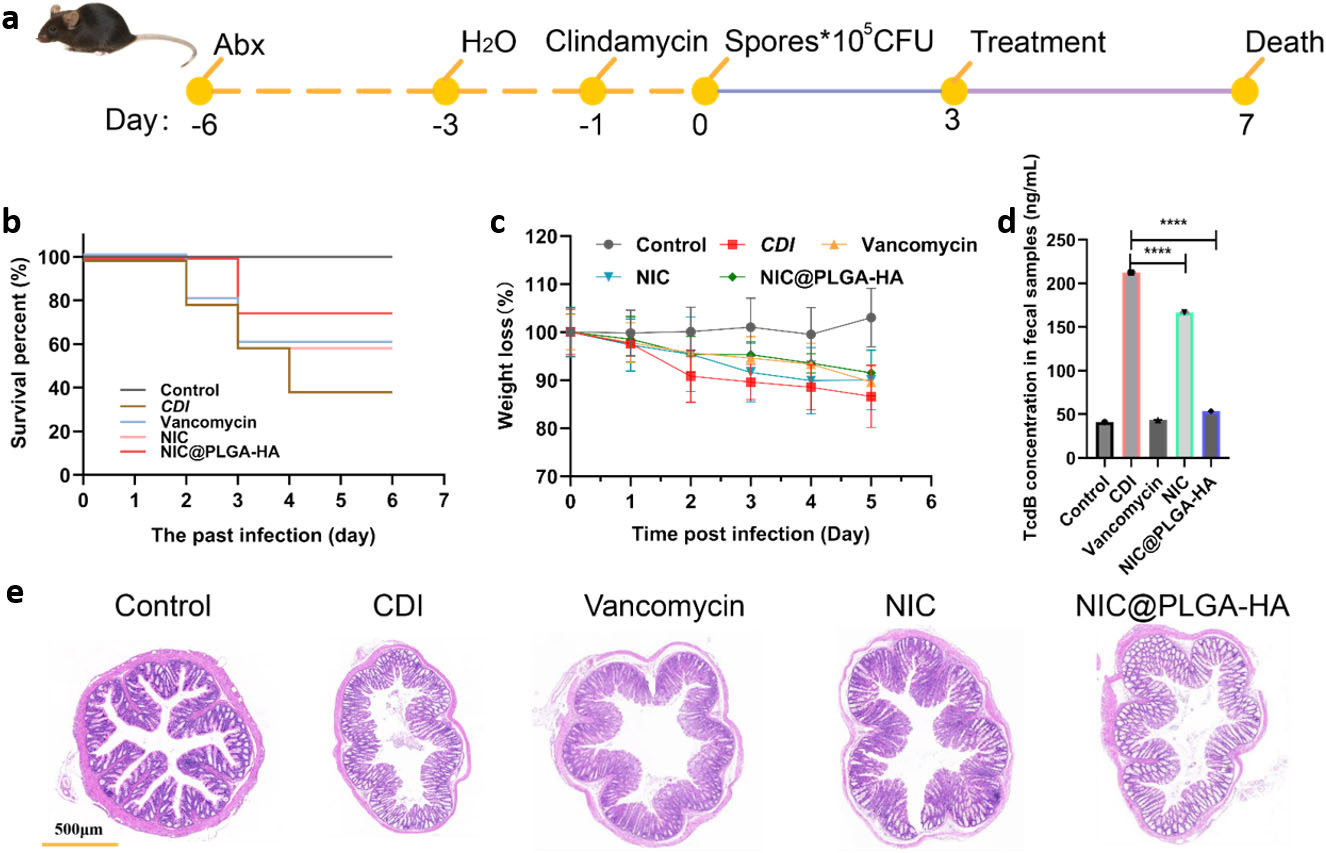
The Mongolian gerbils with *C. difficile* infection (CDI) were treated by NIC@PLGA-HAs. **(A)** Protocol schematic for the Mongolian gerbils CDI model. **(B)** Survival of Mongolian gerbils treated and untreated with vancomycin, Niclosamide (NIC) or NIC@PLGA-HAs after *C. difficile* challenge. **(C)** Weights of Mongolian gerbil after *C. difficile* spore challenge. **(D)** TcdB concentration in fecal samples detected by EILSA (****P < 0.001). **(E)** HE staining of colon tissues from different groups of Mongolian gerbils. Vancomycin was used as a positive control.

Mongolian gerbils challenged with *C. difficile* were treated with NIC, NIC@PLGA-HA, and vancomycin (the clinical CDI therapeutic agent used as positive control), respectively (Figure 5a). NIC and NIC@PLGA-HAs exhibited significant evidence of *in vivo* treatment efficacy in the CDI animal model. Typical CDI symptoms in Mongolian gerbils included severe weight loss on days 2 and 3 post-challenge, accompanied by diarrhea and a high mortality rate (Wan et al., 2024). NIC@PLGA-HAs remarkably protected Mongolian gerbils from death, as indicated by the higher survival rate than those in the vancomycin- and NIC-treated CDI groups (Figure 5b). All the treated animals showed less weight loss than the CDI control group (Figure 5c). NIC@PLGA-HAs inhibited toxin production more effectively than NIC, and was as effective as vancomycin (*P* < 0.001) (Figure 5d). Intestinal histopathological analysis indicated that animals in the CDI group exhibited infection characteristics, including increased inflammatory cell infiltration into intestinal tissues, enhanced mucus secretion, and significant epithelial cell shedding, which were significantly reduced in the NIC@PLGA-treated group (Figure 5e). NIC@PLGA-HAs and vancomycin protected Mongolian gerbils challenged with *C. difficile* at similar levels but via different mechanisms. Vancomycin directly killed *C. difficile* vegetative cells (Darkoh et al., 2022), and however NIC@PLGA-HAs not only inhibited the growth of *C. difficile* but also antagonize TcdB. Free NIC in its ethanolamine salt form (NEN) demonstrated inherent microbiota compatibility, causing no structural disruption and even increasing α-diversity in *C. difficile*-infected mice (Tam et al., 2018). While systemic PLGA delivery can reduce microbial diversity (Chaplin et al., 2020), the hyaluronic acid (HA) component counteracted this effect. Thus, the NIC@PLGA-HA nanocomposites theoretically combined NIC’s virulence-targeted action with HA’s microbiome-stabilizing properties while mitigating PLGA-associated risks, which was worthy of being studied in the later (Mao et al., 2021).

## 4 Conclusion

Our findings revealed that NIC@PLGA-HAs, with high solubility and stability and the pH-dependent nature of drug release, effectively killed *C. difficile* vegetative cells at a MIC of 4 μg/mL and simultaneously inhibited biofilm formation and spore germination. NIC interacted with TcdB at 24 amino acid sites within the DRBD, and however the key sites involved in NIC binding to the TcdB DRBD remained to be determined. Notably, NIC@PLGA-HAs showed superior treatment efficacy over free NIC *in vivo*, with increasing the survival rate and reducing CDI pathological injuries. *In vitro* and *in vivo* studies revealed that NIC@PLGA-HAs exhibited extended-release kinetics compared to free NIC, along with significantly improved aqueous solubility, enhanced chemical stability, and pH-responsive release behavior. These improvements might further improve oral bioavailability of NIC@PLGA-HAs compared to free NIC, and present superior inhibition of *C. difficile* spore germination and biofilm formation. We are also going to investigate synergistic interactions on treatment of CDI led by hypervirulent *C. difficile* strains by combining NIC@PLGA-HAs with vancomycin or fidaxomicin in the future studies. On the other hand, *C. difficile* drug resistance associated gene mutations induced by prolonged NIC@PLGA-HAs exposure should also be further investigated later.

## Data Availability

The original contributions presented in this study are included in this article/Supplementary material, further inquiries can be directed to the corresponding authors.

## Footnotes

1
http://www.rcsb.org/pdb
