# Supplementary material for: Synthetic niclosamide-loaded controlled-release nanospheres with high solubility and stability exerting multiple effects against Clostridioides difficile
Source: Front Microbiol. 2025 Jul 30;16:1617631. doi: 10.3389/fmicb.2025.1617631 (PMC12343512; doi:10.3389/fmicb.2025.1617631)
Supplement: Supplementary file 1 [file Table_1.docx]

Supplementary Material

**Figure Legend**

**Fig. S1.** DTG curves of NIC, PLGA, HA and NIC@PLGA-HAs. Key thermal decomposition events are identified by peak minima: NIC (~307.7°C), PLGA (~324.3°C), HA (~238.2°C), and NIC@PLGA-HAs (~361.7°C). The NIC@PLGA-HAs composite curve demonstrated the thermal behavior of the combined system.

**Fig. S2.** TEM Images of NIC@PLGA-HAs synthesized under varied process parameters. Morphology variations correlate with synthesis conditions, including ultrasonication time (5 min, 10 min), NIC concentration (2.5 mg, 3.0 mg, and 3.5 mg), and ultrasonic power (100 W, 120 W, 150 W, 280 W, and 350 W). These parameters significantly influence both the NIC encapsulation efficiency within the PLGA-HA matrix, and dispersion quality of the resulting NIC@PLGA-HAs.

**Fig. S3.** Effects of (a) power and (b) concentration of NIC on particle diameters and zeta potential of NIC@PLGA-HAs in aqueous solutions. (a) NIC@PLGA-HAs (3.5mg) were synthesized for 5 min under different powers. (b) Different concentrations of the NIC were used to synthesize NIC@PLGA-HAs for 5 min under the 150w ultrasonic wave.

**Fig. S4.** The expression and purification of DRBD^WT^ and DRBD^MT^ by SDS-PAGE. (a) DRBD^WT^: M. Maker; 1. Before induction, lysate, supernatant of *E. coli* BL21 transformed with pET28- DRBD^WT^; 2. Before induction, lysate, precipitation of *E. coli* BL21 transformed with pET28- DRBD^WT^; 3. After induction, lysate, supernatant of *E. coli* BL21 transformed with pET28- DRBD^WT^; 4. After induction, lysate, precipitation of *E. coli* BL21 transformed with pET28- DRBD^WT^; 5. Purified DRBD^WT^ protein; 6. Purified DRBD^WT^ protein; (b) DRBD^MT^: M. Maker; 1. After induction, *E. coli* BL21 transformed with pET28; 2. Before induction, *E. coli* BL21 transformed with pET28- DRBD^MT^; 3. After induction, lysate, supernatant of *E. coli* BL21 transformed with pET28- DRBD^MT^; 4. After induction, lysate, precipitation of *E. coli* BL21 transformed with pET28- DRBD^MT^; 5. Elution buffer; 6. Purified DRBD^MT^ protein.

**Fig. S5.** Views of the interaction between each of TcdB, DRBD, and mutated DRBD and NIC using the AlphaFold 3 (electrostatic surface).

# Supplementary Figure


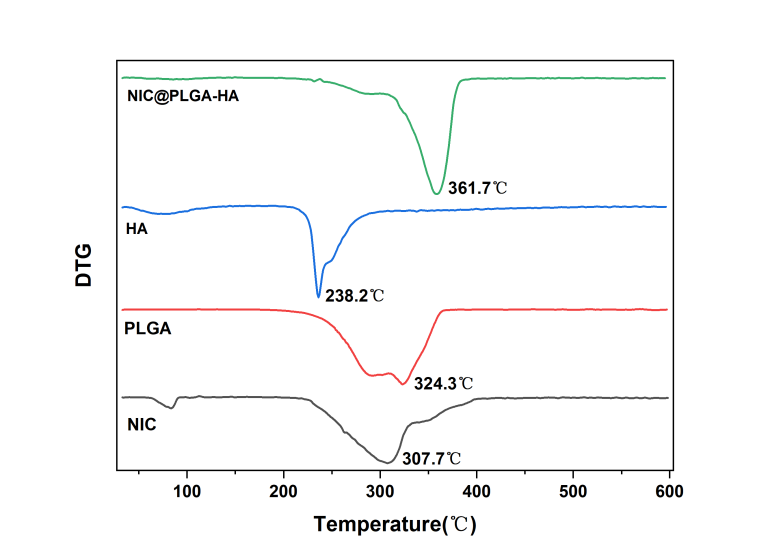


**Fig. S1.**


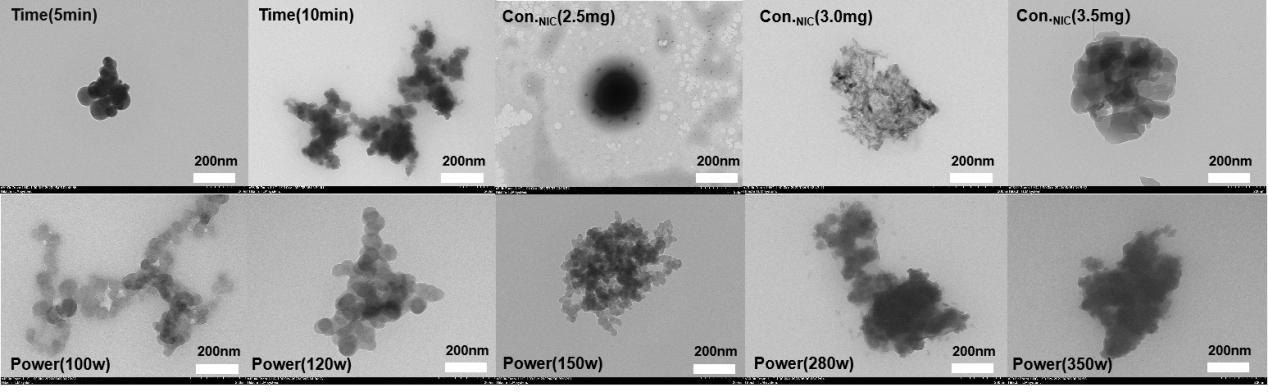


**Fig. S2**


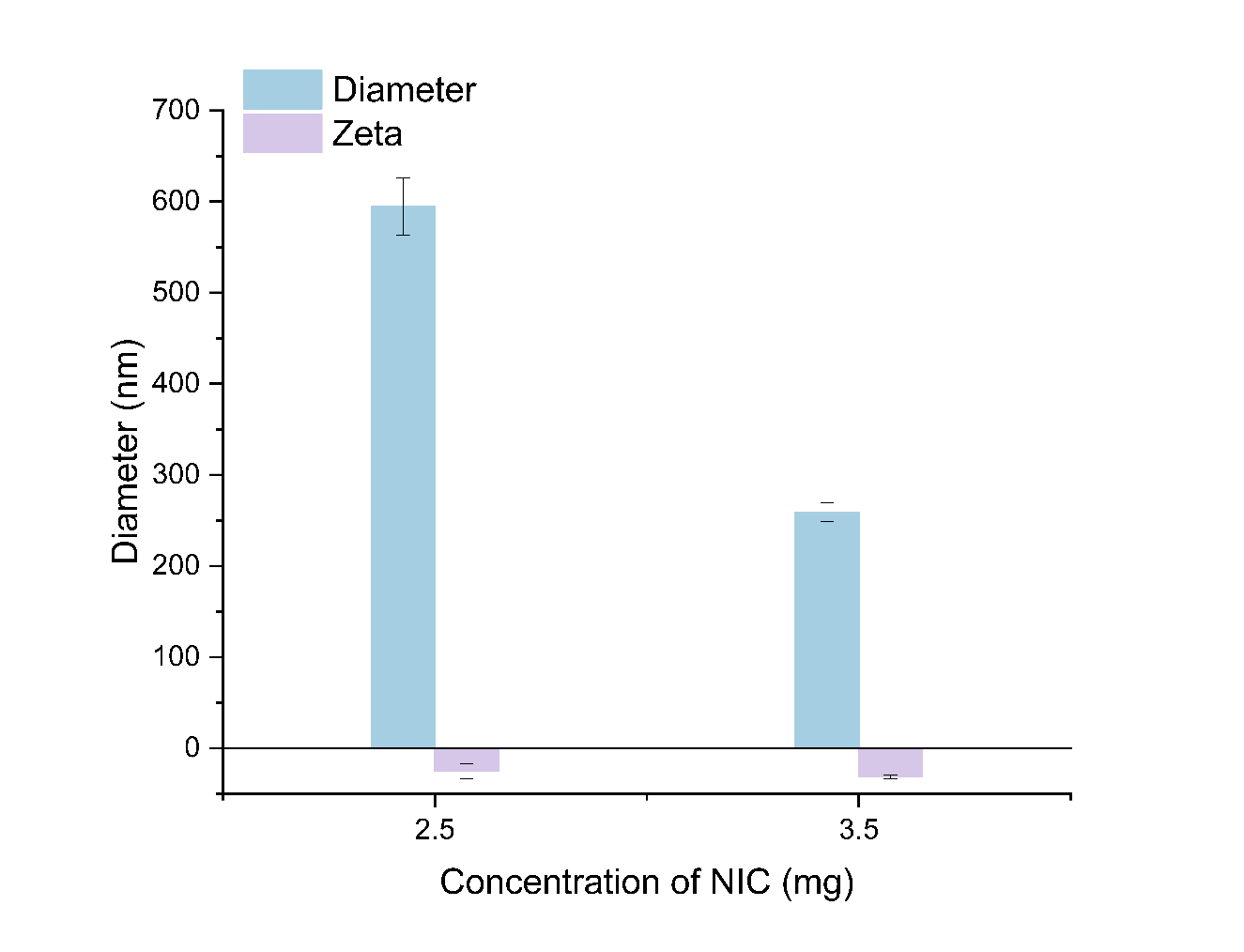

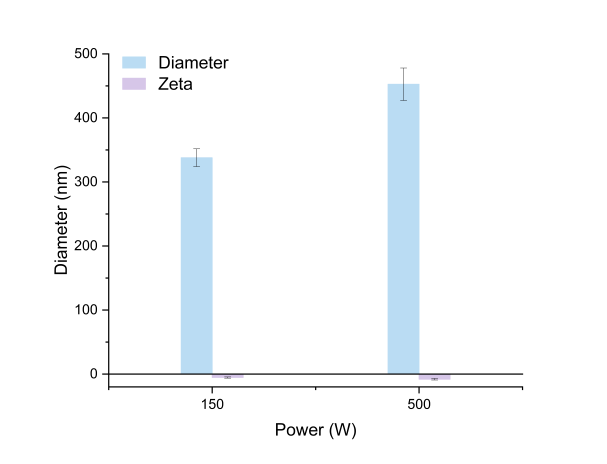


**a**

**b**

**Fig. S3**


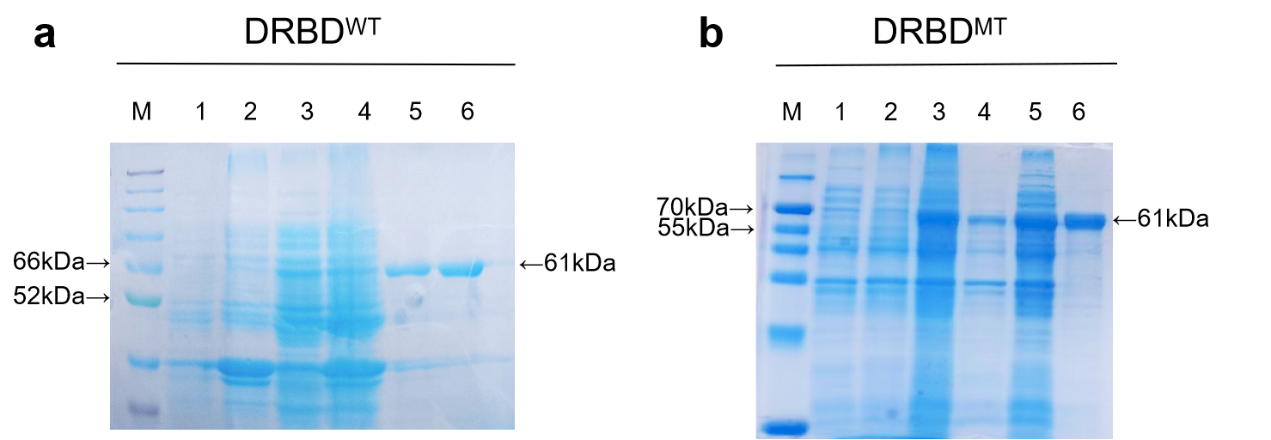


**Fig. S4**

**Binding energy (–6.30 Kcal/mol)**

**Binding energy (–4.10 Kcal/mol)**

| 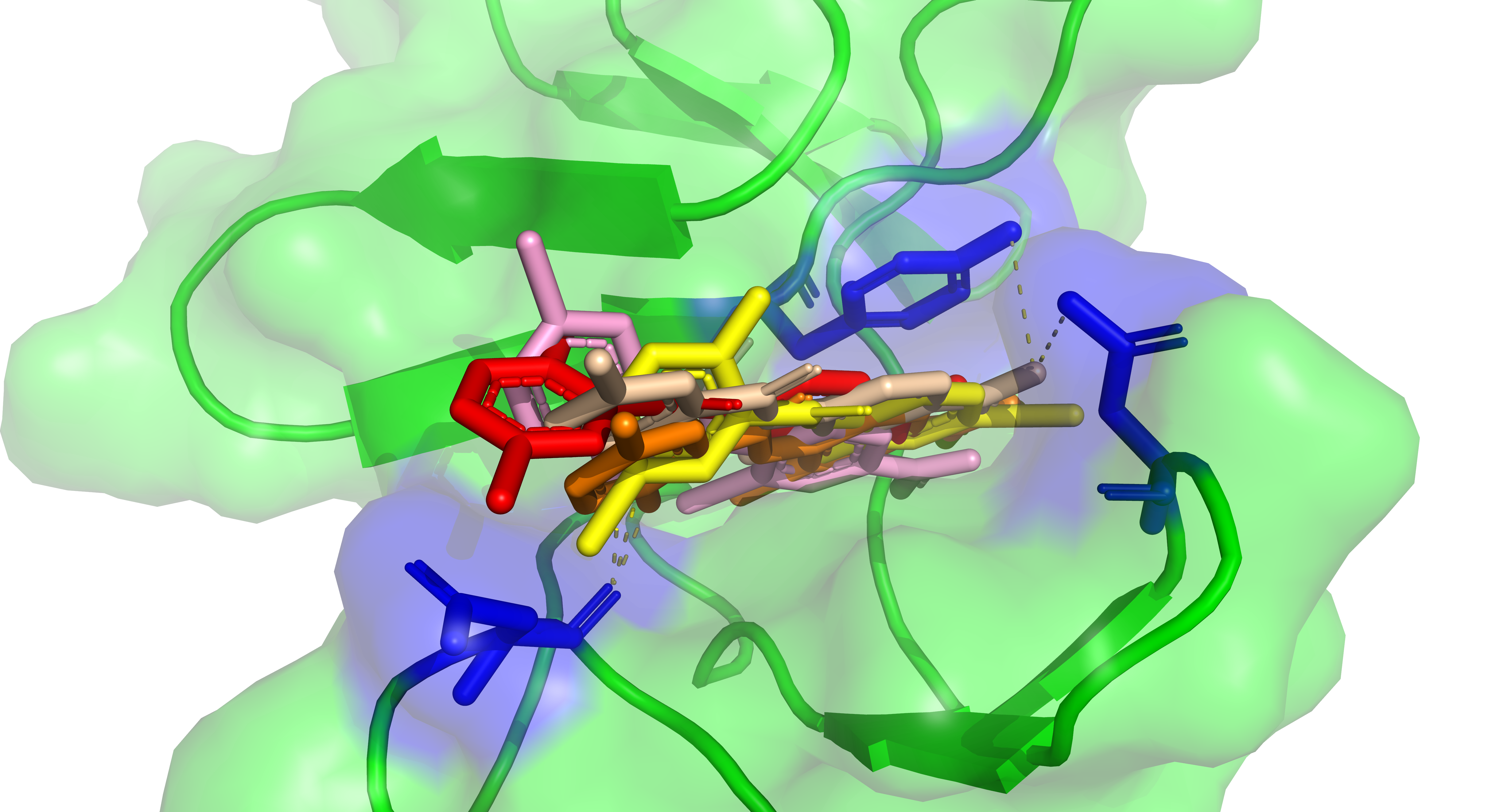  **GLU-2280**  **GLU-2317**  **GLU-467**  **GLU-430** | 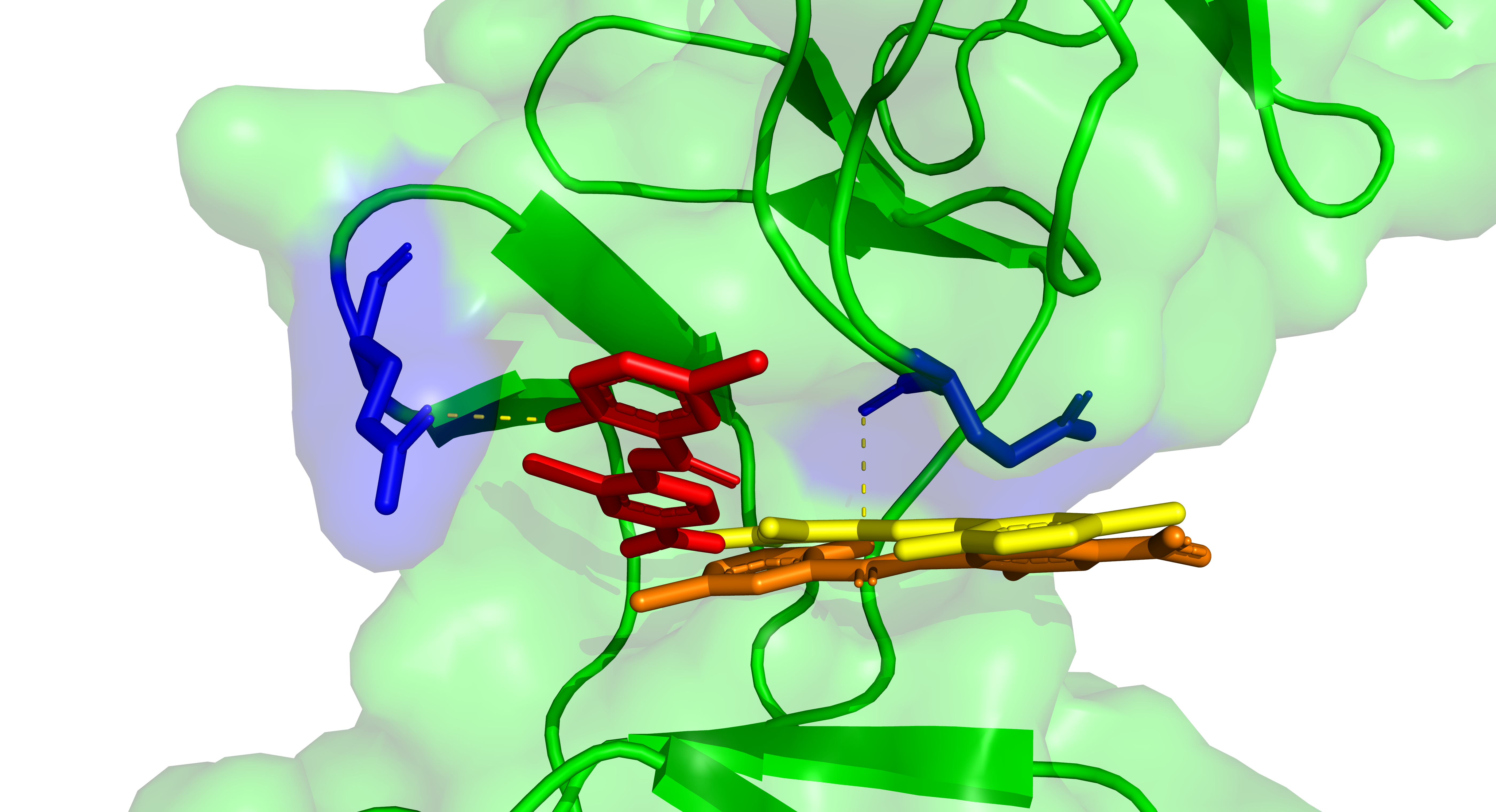  **ASN-2125**  **ASN-2111**  **ASN-2154** |
| --- | --- |

**Fig. S5**

# Supplementary Table

## Table S1. Mutant amino acids in DRBD

| No. | Wild type amino acid | Mutated amino acid | Amino acid site |
| --- | --- | --- | --- |
| 1 | Asn | Met | 8 |
| 2 | Ile | Met | 10 |
| 3 | Tyr | Phe | 44 |
| 4 | Ser | Met | 57 |
| 5 | Thr | Met | 107 |
| 6 | Asp | Glu | 118 |
| 7 | Arg | Met | 121 |
| 8 | Asp | Glu | 158 |
| 9 | Ser | Cys | 159 |
| 10 | Ala | Val | 232 |
| 11 | Lys | Met | 237 |
| 12 | Lys | Met | 265 |
| 13 | Asn | Ile | 282 |
| 14 | Tyr | Asp | 430 |
| 15 | Asp | Glu | 437 |
| 16 | Asn | Met | 454 |
| 17 | Thr | Met | 455 |
| 18 | Lys | Met | 460 |
| 19 | Ser | Gly | 476 |
| 20 | Asn | Tyr | 478 |

| Primer name | Sequences (5'-3') | T_m_/℃ |
| --- | --- | --- |
| DRBD-F | CCATGCATCACCACCACCACCATAATC | 63℃ |
| DRBD-R | TTAGTGGTGGTGGTGATGATGTTCGCT | 63℃ |
| Mutated DRBD-F | TCACCACCACCACTAAGGATCCGAATTCGA | 60℃ |
| Mutated DRBD-R | ATCCGAATTCGAGCTCCGTCGACAAGCTTG | 62℃ |

## Table S2. Primer sequence
